# Metabolic syndrome is not associated to an increased risk of low bone mineral density in men at risk for osteoporosis

**DOI:** 10.1007/s40618-021-01638-w

**Published:** 2021-07-27

**Authors:** D. Rendina, L. D’Elia, G. De Filippo, V. Abate, M. Evangelista, A. Giaquinto, B. Barone, G. Piccinocchi, D. Prezioso, P. Strazzullo

**Affiliations:** 1 Department of Clinical Medicine and Surgery, Federico II University, 80131 Naples, Italy; 2 Assistance Publique—Hôpitaux de Paris, Hôpital Robert-Debré, Service d’Endocrinologie et Diabétologie, 48, Boulevard Sérurier, 75019 Paris, France; 3 Department of Neuroscience Reproductive Sciences and Dentistry, Federico II University, 80131 Naples, Italy; 4 “COMEGEN” Medical Cooperative, 80126 Naples, Italy

**Keywords:** Osteoporosis, Metabolic syndrome, Epidemiological survey, Waist circumference

## Abstract

**Purpose:**

We have recently demonstrated a significant association between osteoporosis (Op) and metabolic syndrome (MetS) in Caucasian women examined by Dual-energy X-ray absorptiometry (DXA) for suspected Op. This cross-sectional study was performed to evaluate the association between MetS and Op in Caucasian men enrolled in the same geographical area, with identical criteria and in the same time range.

**Methods:**

Among subjects enrolled in the SIMON study, we selected the medical records of all free-living men who performed a contextual evaluation of both bone mineral density (BMD) by DXA and MetS constitutive elements (arterial blood pressure, waist circumference, serum levels of triglycerides, high-density lipoprotein cholesterol, and fasting glucose). All enrolled subjects refer to “COMEGEN” general practitioners’ cooperative operating in Naples, Southern Italy.

**Results:**

Overall, the medical records of 880 men were examined. No significant association between MetS and Op was observed. Among MetS constitutive elements, waist circumference was inversely related to Op risk.

**Conclusion:**

In Caucasian men examined by DXA for suspected Op, no significant association was observed between Op and MetS. The study results contrast to those observed in women enrolled in the same geographical area, with identical criteria and in the same time range and may be related to sexual dimorphism occurring in clinical expressiveness of both MetS and Op.

## Introduction

Metabolic syndrome (MetS) is defined by the coexistence of interconnected pathological conditions that increase the risk of type 2 diabetes mellitus (T2DM), cardiovascular diseases (CVD), and mortality for CVDs and for all-causes [1, 2]. Although several medical organizations proposed different MetS diagnostic criteria, the constitutive elements that are considered for MetS definition are elevation of blood pressure and glucose, abdominal obesity, and atherosclerotic dyslipidaemia in all cases [1, 2]. Osteoporosis (Op) is characterized by reduced bone mass, deterioration of bone tissue, and disruption of bone micro-architecture which compromise bone strength and increase the risk of fractures [3–5]. The gold standard criterion for Op diagnosis is the assessment of bone mineral density (BMD) by the dual-X-ray absorptiometry (DXA) [3–5]. Both MetS and Op showed high prevalence and incidence in adult worldwide population and the World Health Organization (WHO) estimations indicate a significant and exponential increase in the incidence and prevalence of MetS and Op in both sexes in the next future caused by the aging of the worldwide populations [2–6]. Based on WHO data, in the last decades, the prevention, detection and treatment of both Op and MetS are considered a priority objective for several public health programs [7, 8]. In this regard, the Italian National Health Service (Sistema Sanitario Nazionale in Italian, SSN) proposes (i) the BMD assessment via DXA to all women and men of any age with an Op major risk factor, and in postmenopausal women and men over the age of 60 years with at least three or more Op minor risk factors, and (ii) the global cardiovascular risk assessment by determination of all MetS constitutive elements to all women and men over the age of 39 years [9, 10]. The SIMON (SIndrome Metabolica, Osteoporosi e Nefrolitiasi in Italian; metabolic syndrome, osteoporosis and nephrolithiasis) study is an epidemiological survey based on administrative data performed in Naples, Southern Italy, to estimate the relationship occurring between all these frequent and multifactorial disorders [11, 12]. Thanks to the SIMON study protocol, we have recently demonstrated a significant association between Op and MetS in free-living Caucasian women referred to DXA for suspected Op [11]. Considering that all the aging-related diseases, including Op and MetS, show an evident sexual dimorphism in their clinical expressiveness [13], we used the SIMON database to perform a cross-sectional study finalised to evaluate if MetS and its constitutive elements are associated to an increased Op risk also in free-living Caucasian men with suspected Op who underwent DXA examination.

## Methods

The SIMON study protocol has been extensively previously described [11, 12]. Briefly, the SIMON database is constituted by the medical records of 180,724 patients referring to the general practitioners (GPs) registered to the “COMEGEN” Medical Cooperative on June 1, 2018. For this cross-sectional study, the COMEGEN GPs selected Caucasian men who performed contemporarily the BMD assessment by DXA [International Classification of Diseases—9th revision (ICD9) code 8898] and the evaluation of the presence of MetS constitutive elements, according to the American Heart Association (AHA) /National Heart, Lung, and Blood Institute (NHLBI) Scientific Statement [14]. The COMEGEN GPs examined the time interval comprised between June 1, 2008 and May 31, 2018. On their first admission to their GPs, all patients accepted that (i) their personal data could be used for public health research purposes; (ii) they could withdraw their consent to the use of personal data without any explanation at any time and without effects on the medical assistance. This, in the form of written informed consent, was obtained from each patient or subject involved in this study. The study protocol was approved by the ASL Naples 1 Ethical Committee, protocol number 0018508/2018.

### Diagnostic criteria

The Op diagnosis was made in men showing at least one of these criteria (i) a DXA T-score value ≤ − 2.5 in the lumbar spine, total hip or femoral neck [15]; (ii) personal history of clinical fragility fractures and/or anti-osteoporotic treatment independent of BMD values. Men without any diagnostic criteria were considered as controls. The MetS diagnosis was made in Caucasian men showing at least any three of the five following criteria: (1) waist circumference ≥ 102 cm; (2) serum triglycerides > 1.7 mmol/L or current drug treatment for elevated triglycerides; (3) serum High-Density Lipoprotein cholesterol (HDL cholesterol) < 1.03 mmol/L or drug treatment for dyslipidemia; (4) systolic blood pressure (BP) ≥ 130 mmHg and/or diastolic BP ≥ 85 mmHg or current antihypertensive drug treatment in a patient with a history of hypertension and (5) fasting serum glucose ≥ 5.6 mmol/L or drug treatment for T2DM [14]. According to Grundy and colleagues [14], to measure the waist circumference, the top of right iliac crest was located. A measuring tape was placed in a horizontal plane around abdomen at level of iliac crest. Measurement is made at the end of a normal expiration ensuring that, before reading tape measure, the tape is snug but does not compress the skin and is parallel to floor.

### Exclusion criteria

The clinical records of Caucasian men with age lower than 40 years, long-term immobilization, regular use of gonadotropin-releasing hormone agonist, glucocorticoids, anticonvulsants, heparin, vitamin A, cytotoxic agents and antiandrogens, Op therapy not compliant to Italian Medicine Agency (Agenzia Italiana del FArmaco, AIFA) prescriptive criteria [16], DXA prescription not compliant to Italian Essential Assistance Levels (EAL) [9, 10], clinical conditions reported in Table 1 and incomplete data collection were excluded from this study.

**Table 1:** Clinical conditions considered exclusion criteria for patients’ enrolment

Clinical conditions	ICD9 codes
Malabsorption syndromes	From 5793 to 5799
Rheumatoid arthritis	7140
Moderate to severe CKD	From 5853 to 5859, 586 and 6393
Hyperthyroidism	From 24,200 to 24,291
Primary hyperparathyroidism	From 25,200 to 25,208
Hypoparathyroidism	2521
Cushing’s syndrome	2550
Chronic liver disease	From 5710 to 5719
Pituitary tumors	1943, 2273, 2370
Surgical history of terminal ileal resection	4562
Surgical history of gastrectomy or small bowel bypass	From 430 to 4499
Eating disorders	3071, from 30,750 to 30,759
Alcoholism	From 30,390 to 30,393

*ICD9* International Classification of Diseases 9th revision, *CKD* chronic kidney disease

### Statistical analysis

All statistical analyses were performed using the IBM SPSS Statistics software, version 23 (International Business Machines Corporation, Armonk, New York) by L.D.E., who did not participate to the data extraction from “COMEGEN” Medical Cooperative database. Analysis of variance (ANOVA) or Chi-squared test was used to assess differences in the main characteristics of men who had or had not Op and/or MetS. Binary logistic regression analysis was used to estimate the influence of MetS on the risk of Op, adjusting for the main potential confounders (Model 1: age and drugs reported in Table 2; Model 2: age, drugs reported in Table 2, and numbers of MetS constitutive elements; Model 3: age, drugs reported in Table 2, and the individual MetS constitutive elements). The results are reported as mean and standard deviation (SD) as percentages or as odds ratio (OR) and 95% confidence intervals (95% CI), unless otherwise indicated. All reported p values are two-sided, and the significant level was set at *p* < 0.05.

**Table 2:** Use of drugs associated to reduced BMD in SIMON study cohorts

Drugs	Op (*n* = 552)	Controls (*n* = 328)	*p*
Proton pump inhibitors (*n*; %)	338; 61.2	190; 57.9	0.36
Thiazolidinediones (*n*; %)	20; 3.6	16; 4.9	0.38
Antiretroviral therapy (*n*; %)	54; 9.8	30; 9.1	0.81
Loop diuretics (*n*; %)	92; 16.7	50; 15.2	0.64
Calcineurin inhibitors (*n*; %)	4; 0.7	2; 0.6	0.98

Data are expressed as absolute; percentage number

*p* values were determined using Chi-squared test

*BMD *bone mineral density. *SIMON* sindrome metabolica, osteoporosi e nefrolitiasi in Italian; metabolic syndrome, osteoporosis and nephrolithiasis. *Op* SIMON men with clinical diagnosis of osteoporosis. *Controls* SIMON men without clinical diagnosis of osteoporosis

## Results

According to exclusion and inclusion criteria previously exposed, the medical records of 880 Caucasian men [mean age 73.5 ± 9.0 years; range 42–90 years Body Mass Index (BMI) 26.9 ± 4.1 kg/m^2^] were selected and examined. Five hundred fifty two have Op clinical diagnosis (Op patients; mean age 73.9 ± 9.0 years; BMI 26.6 ± 4.1 kg/m^2^), whereas the remaining 328 men without Op diagnosis were considered as controls (mean age 72.7 ± 8.8 years; BMI 27.6 ± 4.0 kg/m^2^). Op patients showed a significantly lower BMI compared to controls (*p* < 0.01).

Overall, 827 men had hypertension or current antihypertensive treatment (mean age 73.9 ± 8.7 years; BMI 27.0 ± 4.1 kg/m^2^; 518 with Op), 609 subjects had high fasting serum glucose or T2DM (mean age 73.3 ± 8.9 years; BMI 27.4 ± 4.3 kg/m^2^; 375 with Op), 716 men had low serum HDL cholesterol or current drug treatment (mean age 73.5 ± 8.7 years; BMI 27.1 ± 4.0 kg/m^2^; 450 with Op), 707 men had high serum triglycerides or current drug treatment (mean age 73.3 ± 8.6 years; BMI 27.1 ± 4.1 kg/m^2^; 443 with Op), 436 men had high waist circumference (mean age 73.7 ± 9.2 years; BMI 30.1 ± 3.1 kg/m^2^; 248 with Op). Twenty men (mean age 65.1 ± 11.0 years; BMI 23.8 ± 1.5 kg/m^2^; 17 with Op) did not have any MetS constitutive element, 40 men (mean age 74.0 ± 10.3 years; 23.6 ± 2.2 kg/m^2^; 24 with Op) had a single MetS constitutive element, while 76 men (mean age 75.9 ± 9.3 years; BMI 26.0 ± 3.7 kg/m^2^; 43 with Op) had two MetS constitutive elements. The remaining 744 men have MetS showing at least three MetS constitutive elements (mean age 73.4 ± 73.8 years; BMI 27.3 ± 4.1 kg/m^2^): of these, 468 (63%; mean age 74.0 ± 8.7 years; BMI 26.9 ± 4.1 kg/m^2^) had Op diagnosis.

In the study population, Op and MetS were not significantly associated (OR 1.04; 95% CI 0.71–1.51; *p* = 0.80) and the Op prevalence did not increase, increasing the numbers of MetS constitutive elements (*p* for trend = 0.06). In a separate logistic regression model including the individual components of MetS (Table 3), waist circumference was the only MetS constitutive elements related to the occurrence of Op, accounting for age, all the other MetS constitutive elements, and ongoing treatments.

**Table 3:** Risk of osteoporosis and metabolic syndrome constitutive elements in study cohort

MetS constitutive elements	Odds ratio	95% confidence interval	*p*
Hyperglycaemia/T2DM	0.90	0.66–1.24	0.50
Hypertension	0.91	0.48–1.68	0.78
Low HDL cholesterol levels	1.14	0.60–2.14	0.72
Hypertriglyceridaemia	0.99	0.54–1.91	0.99
High waist circumference	0.60	0.47–0.80	< 0.01

Hyperglycaemia/T2DM: fasting serum glucose ≥ 5.6 mmol/L or drug treatment for T2DM; Hypertension: systolic blood pressure (BP) ≥ 130 mmHg and/or diastolic BP ≥ 85 mmHg or current antihypertensive drug treatment in a patient with a history of hypertension; Low HDL cholesterol levels: serum HDL cholesterol < 1.03 mmol/Lor drug treatment for dyslipidemia. Hypertriglyceridemia: serum triglycerides > 1.7 mmol/L or current drug treatment for elevated triglycerides; High waist circumference: waist circumference ≥ 102 cm; The MetS diagnosis was made in men who fulfilled any three of the previously exposed criteria [14]

*MetS* metabolic syndrome; *T2DM*: type 2 diabetes mellitus; *HDL* high-density lipoprotein

## Discussion

The study results do not demonstrate a significant association between MetS and Op in free-living Caucasian men from Southern Italy undergoing to DXA for suspected Op. This study is part of the SIMON study protocol [11, 12]. Within the SIMON protocol, we have recently performed a study aimed to evaluate the relationship between MetS and Op in free-living women at risk of osteoporosis, according to the Italian EAL [11]. Both men and women enrolled in the SIMON study live in the same geographic area and were enrolled in the same time laps [11, 12]. Unlike what was observed in SIMON men, a significant association between MetS and Op was observed among SIMON women [11]. However, the SIMON women cohort is larger (13,182 participants) and younger (mean age 62.8 ± 9.4 years) compared to SIMON men cohort. These differences prompted a separate analysis of the two cohorts and may justify the different clinical characteristics of men and women enrolled in the SIMON study [11]. In effect, the prevalence of MetS (84.5% vs. 59.3% for men and women, respectively) and Op (62.7% vs. 84.4% for men and women, respectively) is different in SIMON men and SIMON women [11]. Also, the prevalence of high blood pressure/hypertension (94.0% vs. 84.1% for men and women, respectively), high serum triglycerides (80.3% vs. 56.5% for men and women, respectively), high fasting serum glucose and/or T2DM (69.2% vs. 28.1% for men and women, respectively), low HDL cholesterol (81.4% vs. 54.4% for men and women, respectively) and high waist circumference (49.5% vs. 66.3% for men and women, respectively) is significantly different in SIMON men and SIMON women [11]. MetS and Op are two major public health problems with enormous human, social, and financial cost. Both disorders showed a similar and complex pathogenesis characterized by the interaction between non-modifiable and modifiable risk factors, such as unhealthy dietary habits and low physical activity [7, 8]. Even disorders of vitamin D and calciotropic hormones may be implicated in the pathogenesis of both disorders [17, 18]. Epidemiological surveys however demonstrated that both MetS and Op are characterized by a sexual dimorphism in their clinical expressiveness [13]. In effect, the Op prevalence is significantly higher in women than in men; in 2010, there were 22 million women and 5.5 million men affected by Op in Europe [19, 20]. From a clinical point of view, men show a lower lifetime fracture risk compared to women (13–25% vs 50%) [21]. In absolute numbers, the prevalence of vertebral or hip fracture in elderly men is approximately one-third of that in women, but on the contrary, the mortality rate associated with hip fractures, as well as vertebral and other major fractures, is higher in men than in women [22–25]. Regarding MetS, a large population-based project enrolling 36 cohorts from 10 European countries (the MOnica, Risk, Genetics, Archiving and Monograph—MORGAM project) [26] found that the prevalence of MetS was more than doubled in women when compared to men in a population aged 19–39 to 60–78 years old. When individually analysing the patterns of MetS components, men showed a higher prevalence of arterial hypertension and hypertriglyceridemia, while women had more frequently increased waist circumference and low HDL cholesterol levels. According to these results, Dallongeville and colleagues demonstrated that elevated waist circumference and low HDL cholesterol were significantly larger contributors to MetS in women than in men, while hypertension was the metabolic disorder most frequently found in men than in women [27]. These phenomena may be linked to different physiological role of mitochondrial malfunction and abnormal mitochondrial-nuclear signalling and of sexual hormones in the two genders through the lifespan from birth to senescence [13, 28–30]. Analysing the influence of each MetS constitutive element on Op diagnosis, the study results indicate that higher waist circumference is associated to a reduced risk of low BMD in men at increased risk of osteoporosis according to the Italian EAL. This association was independent of others MetS constitutive elements. Different studies analysed the associations between some adiposity indices, including waist circumference, and BMD or osteoporosis [31–35]. These studies were preferentially performed in women and furnish contradictory findings, probably for the different inclusion criteria of covariates used in the statistical models used for evaluate the possible association. The mechanisms proposed to explain the inverse association between obesity and BMD are essentially two. The obese subjects may have higher BMD for the elevated mechanical load of lean and fat mass on the bones and/or for the anabolic stimulus provided on bone formation by some adipocytokines, in particular adiponectin and resistin [36–39].

### Strength and limits

The SIMON study is based on an administrative database, a study methodology showing well-defined strengths and limits [40]. Certainly, the Op diagnosis based upon administrative health database has an acceptable level of sensitivity, specificity, and accuracy, especially when the assumption of anti-osteoporotic drugs was used as additional information in the query formulation, as in our study [41]. Also, MetS diagnostic criteria used in SIMON protocol are suitable for epidemiological studies [2] and have been previously used in studies with similar characteristics [11, 42, 43]. In this regard, it should be noted that in 2009, some important health organizations, including AHA and NHLBI, met to draft a Joint Scientific Statement (JSS) with the intention of harmonizing the MetS diagnosis worldwide [44]. Not surprisingly, a large part of the JSS is devoted to discussing the diagnosis of abdominal obesity. As admitted in the JSS, the definition of the cut-off points for abdominal obesity is complicated by several factors, including ethnic origin. For Caucasian subjects, a waist circumference ≥ 102 cm in men correspond to a BMI ≥ 30 kg/m^2^ [45]. Finally, we enrolled a large and homogeneous population. On the other hand, the limits related to use of administrative data should be declared. We are unable (a) to evaluate if the anatomical site of DXA exam furnishing pathological BMD finding (i.e. lumbar, femoral or ulnar site) influences the study results, (b) to estimate the role of metabolic and dietary risk factors for both Op and MetS in the observed results, and (c) to improve the quality of available data evaluating the trabecular bone score and the occurrence of vertebral fractures using vertebral morphometry. A crucial point is the generalizability of study results. In effect, the study cohort is representative for all free-living adult men (i.e. over 40 years old), without pathology associated with secondary Op, undergoing DXA evaluation of BMD basing on Italian EAL and not for the entire Italian adult male free-living population. The selection criteria also account for the high prevalence of Op and MetS constitutive elements observed in the study cohort.

In conclusion, the present study demonstrates that MetS is not associated to an increased Op risk in Caucasian men in whom skeletal health was evaluated by DXA exam according to Italian EAL. The comparison of the results of the present study to those obtained in women enrolled in the same geographical region and in the same lapse of time suggests the occurrence of a sexual dimorphism in the clinical expressiveness of MetS and Op. Further ad hoc studies are necessary to confirm this intriguing hypothesis.

## Data Availability

The datasets generated during and/or analysed during the current study are available from the corresponding author on reasonable request.
